# Spiral Field Generation in Smith-Purcell Radiation by Helical Metagratings

**DOI:** 10.34133/2019/3806132

**Published:** 2019-02-27

**Authors:** Liqiao Jing, Zuojia Wang, Xiao Lin, Bin Zheng, Su Xu, Lian Shen, Yihao Yang, Fei Gao, Min Chen, Hongsheng Chen

**Affiliations:** ^1^Key Lab. of Advanced Micro/Nano Electronic Devices & Smart Systems of Zhejiang, College of Information Science and Electronic Engineering, Zhejiang University, Hangzhou 310027, China; ^2^Department of Physics, Massachusetts Institute of Technology, Cambridge, MA 02139, USA; ^3^School of Information Science and Engineering, Shandong University, Qingdao 266237, China; ^4^Division of Physics and Applied Physics, School of Physical and Mathematical Sciences, Nanyang Technological University, Singapore; ^5^State Key Laboratory of Integrated Optoelectronics, College of Electronic Science and Engineering, Jilin University, Changchun 130012, China

## Abstract

Moving electrons interacting with media can give rise to electromagnetic radiations and has been emerged as a promising platform for particle detection, spectroscopies, and free-electron lasers. In this letter, we investigate the Smith-Purcell radiation from helical metagratings, chiral structures similar to deoxyribonucleic acid (DNA), in order to understand the interplay between electrons, photons, and object chirality. Spiral field patterns can be generated while introducing a gradient azimuthal phase distribution to the induced electric dipole array at the cylindrical interface. Experimental measurements show efficient control over angular momentum of the radiated field at microwave regime, utilizing a phased electromagnetic dipole array to mimic moving charged particles. The angular momentum of the radiated wave is determined solely by the handedness of the helical structure, and it thus serves as a potential candidate for the detection of chiral objects. Our findings not only pave a way for design of orbital angular momentum free-electron lasers but also provide a platform to study the interplay between swift electrons with chiral objects.

## 1. Introduction

Angular momentum including spin and orbital angular momentum is a fundamental physical quantity in both classical and quantum physics. The spin angular momentum of light is associated with light polarization, experimentally demonstrated by Beth in 1935 [1], while, until 1992, light carrying orbital angular momentum called optical vortices was firstly investigated by Allen [2] and soon verified experimentally [3]. Since then, various studies on optical vortices have been reported. Special characteristics of optical vortices have attracted plenty of interests from many communities, such as optical tweezers, optical communications, biology microscopy, and so forth [4–6]. Owing to the fascinating properties and wide applications, generating optical vortices has been under intense study and various schemes have been proposed such as spiral phase plate [7], computer generated holograms (CGH) [8], metamaterials/metasurfaces [9–12], and spoof plasmonics [13–15]. Recently, a new approach which is able to produce vortices based on radiation emission in extreme ultraviolet (XUV) even X-ray from electron vortex beams has developed rapidly [16, 17]. Electron vortices have also been explored as detectors of chirality in crystals [18] and molecules [19]. Common schemes of electron vortex beams use spiral phase plates [20–22] or interaction of the e-beam in free-electron lasers (FELs) to the magnetic field of the helical undulator [23–27]

Electron plays a significant role in physics. In particular, swift electrons carry the evanescent field, which can generate far-field radiation when the electrons interact with materials [28]. For example, Cherenkov radiation [29, 30] is emitted when the speed of an electron is greater than that of light in the background medium. Especially, due to the emergence of negative-index metamaterials, reversed Cherenkov radiation has been proposed and experimentally observed [31, 32]. Unlike Cherenkov radiation, Smith-Purcell radiation is generated from the induced current varying in space and time when charges moving near the periodically deformed surface [33, 34]. Recently, the study of Smith-Purcell radiation was extended beyond the simple periodic structure, into aperiodic arrays [35], disordered plasmonic arrays [36, 37], and Babinet metasurfaces [38–40] for manipulation on the Smith-Purcell radiation polarization, beyond the simple periodic structure. However, to the best of our knowledge, few works discuss how to introduce angular momentum into the Smith-Purcell radiation so far.

In this letter, we propose a new method to generate angular momentum in Smith-Purcell radiation when a swift electron passes through a helical metagrating. Semitheoretical analysis of the spiral field generation in Smith-Purcell radiation from a helical metagrating is performed. The intrinsically nonradiative energy bound at the source current sheet is coupled to an electric dipole with an azimuthal phase factor exp⁡(*ilφ*) when swift electrons pass through the helical metagrating. Here, the angular momentum* l* is an integer of two possible values (±1). Subsequently, a circular waveguide filling with dielectric is designed to model moving electrons, which enables the verification of the spiral field generation in Smith-Purcell radiation in the microwave regime. Based on the Smith-Purcell emission [34], our results may find applications in tunable and high power terahertz sources, particle detectors, electron-assisted spectroscopy of chiral matters, and other novel photonic devices.

## 2. Results and Discussion

**Figure 1:**
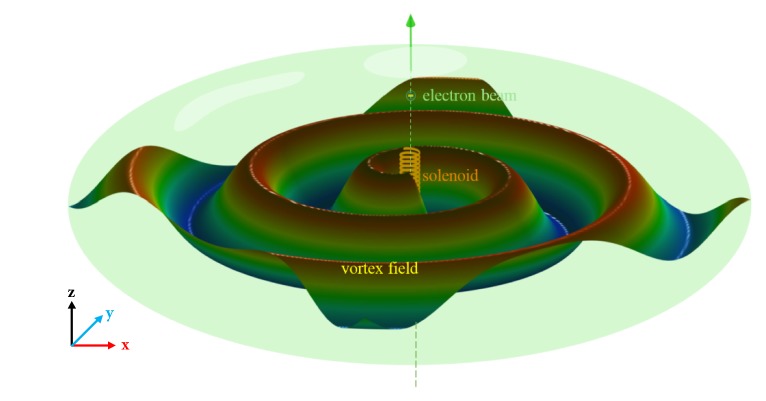
Schematic of spiral field generation in Smith-Purcell radiation with a swift electron passing through a helical metagrating.

The schematic of spiral field generation in Smith-Purcell radiation is illustrated in Figure 1. We consider a swift electron with charge* q*, moving with velocity ${\hat{v}}_{0}=\hat{z}v_{0}=\hat{z}\beta c$ through a helical metagrating, where c is the speed of light in free space and *β* is velocity divided by the speed of light (*β* = v_0_/*c*). When the electron beam moves along the center of helical metagrating, the induced current on the metallic surface will generate spiral field Smith-Purcell radiation if the helix pitch is properly designed. Next, we investigate the necessary conditions to design the helical metagrating. Space-time dependence of current densities is described as $\overset{-}{J}(\overset{-}{r},t)=\hat{z}qv_{0}\delta (x)\delta (y)\delta (z-vt)$, in which an electron goes through the origin at* t* = 0. After transforming to the frequency domain, we obtain

(1) $$\begin{array}{c} \overset{-}{J}\left(\;\overset{-}{r},\omega \;\right)=\frac{1}{2\pi }\int dt\overset{-}{J}\left(\;\overset{-}{r},t\;\right)e^{i\omega t}=\hat{z}I_{0}e^{ik_{z}z} \end{array}$$

where *k*_*z*_ = *ω*/*v*_0_ = *ω*/*βc* and *I*_0_ = *q*/2*π*. In outer space of the helical metagrating, the radiated field can be described in terms of Floquet modes as

(2) $$\begin{array}{c} {\overset{-}{E}}_{r}=\underset{m}{\sum }{\overset{-}{E}}_{m}e^{i\left(k_{z}+2m\pi /p\right)z+ik_{\rho m}\rho }e^{il\varphi } \end{array}$$

Here, $k_{\rho m}=\sqrt{k_{0}^{2}-(k_{z}+2m\pi /p{)}^{2}}$ is wave number in *ρ* direction,* p* is the helix pitch in* z*-direction and *p* < *λ*_0_, and ${\overset{-}{E}}_{m}$ is electric field vector of each diffraction order. To guarantee *k*_*ρm*_^2^ > 0, the necessary condition for Smith-Purcell radiation

(3) $$\begin{array}{c} \frac{p}{\left|m\right|}\left(\;\frac{1}{\beta }-1\;\right)\leq \lambda_{0}\leq \frac{p}{\left|m\right|}\left(\;\frac{1}{\beta }+1\;\right),\;with\;\;m<\text{0}. \end{array}$$

For simplicity, we assume *k*_*z*_ = 0 and the helix pitch is designed as *p* = −*mβλ*_0_, and the radiated wave propagates only along the *ρ* direction (in the* xy*-plane). Compared with the OAM beams in conventional optical vortex generators, the radiated propagation direction of the spiral field from Smith-Purcell radiation is perpendicular to the moving direction of electrons (i.e., the* x*-*y* plane in Figure 1).

**Figure 2:**
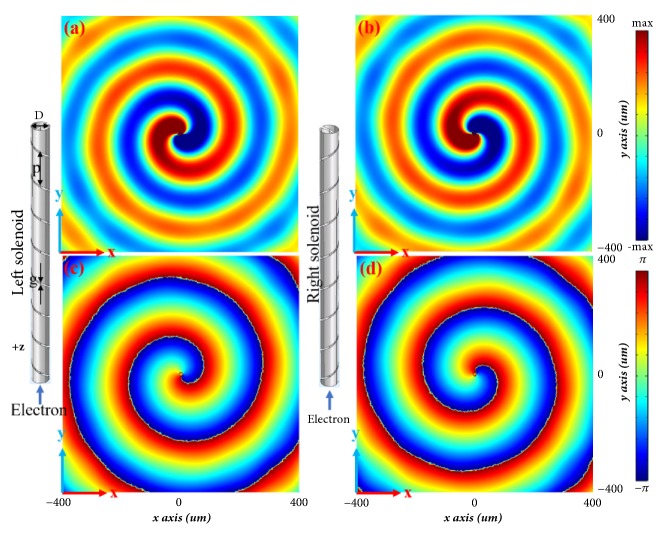
Simulation results of* E*_*z*_ field distributions of the spiral field Smith-Purcell radiation with the angular momentum* l* = ∓1. (a) and (c) Radiated* E*_*z*_ field distribution at* z* = 0 (a) and the corresponding phase distribution for* l* = -1 (c). (b) and (d) Radiated* E*_*z*_ field distribution at* z* = 0 (b) and the corresponding phase distribution for* l* = +1 (d). The working frequency is 1.5 THz.

A proof-of-concept helical metagrating for spiral field generation in Smith-Purcell radiation is designed in the terahertz regime. The diameter of the helical metagrating is* D* = 10* um*, the helix pitch is* p* = 25* um*, and the gap is* g* = 2.5* um*. Gold is chosen as the metallic material with a conductivity of 4 × 10^7^* S/m* and a thickness of 0.1* um*. Full-wave simulations have been performed in commercial software, COMSOL MULTIPHYSICS. In the simulation, a swift electron moves along the +z direction in the center of the helical metagrating. We set the electron velocity as *v*_0_ = *βc* = *c*/8, and the current density as *I*_0_ = 1  *A*/*m*. The periodic boundary condition is imposed in the* z*-direction. Under these conditions, the spiral field Smith-Purcell radiation at 1.5 THz propagates along *ρ* direction when *k*_*z*_ = 0. All field images are* xy*-plane snapshots of electric fields at the position of* z* = 0. When the swift electron passes through a left-handed helical metagrating, spiral field Smith-Purcell radiation is generated with the angular momentum* l = -*1.* E*_*z*_ field distribution and its phase response are plotted in Figures 2(a) and 2(c). On the contrary, for right-handed helical metagrating, the angular momentum is* l = +*1, as shown in Figures 2(b) and 2(d). Furthermore, the phase changes at the* xy*-plane equal to 2*π*, in agreement with the theoretical prediction.

**Figure 3:**
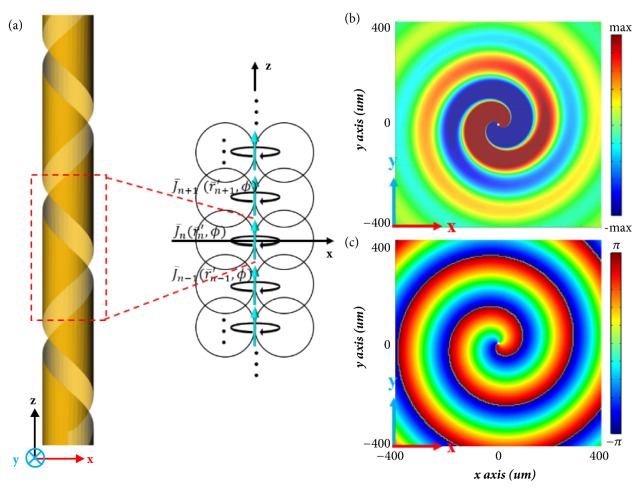
Theoretical equivalent model of the helical metagrating and the calculated radiation fields. (a) Equivalent electric dipole array of the spiral field Smith-Purcell radiation for the left-handed helical metagrating. (b) Calculated* E*_*z*_ field distribution and the (c) corresponding phase distribution at 1.5 THz.

In this section, the underlying physical mechanism is further discussed by adopting a semianalytical analysis. When a swift electron moves through a helical metagrating, the induced current distribution on the metal surface can be modeled as an electric dipole array with an azimuthal phase factor exp⁡(*ilφ*). Each unit cell is considered as an equivalent electric dipole with an azimuthal factor as shown in Figure 3(a). The current density ${\bar{J}}_{n}({\bar{r}}^{\prime },\phi )$ of the* n-th z*-direction Hertzian dipole located at the origin is

(4) $$\begin{array}{c} {\bar{J}}_{n}\left(\;{\bar{r}}^{\prime },\phi \;\right)=\hat{z}Il\delta \left(\;{\bar{r}}^{\prime }\;\right)e^{il\phi } \end{array}$$

The electric field due to the Hertzian dipole is calculated by utilizing Green's function

(5) E¯nr¯,ϕiωμI¯¯+1k2∇∇·∭dr¯′eikr¯−r¯′4πr¯−r¯′J¯nr¯′,ϕ=iωμI¯¯+1k2∇∇·eikr4πrf¯θ,ϕ

where $\bar{f}(\theta ,\phi )=∭d{\bar{r}}^{\prime }{\bar{J}}_{n}({\bar{r}}^{\prime },\phi )e^{-i\bar{k}\cdot {\bar{r}}^{\prime }}=\hat{z}Il\delta ({\bar{r}}^{\prime })e^{il\phi }=(\hat{r}\cos\theta -\hat{\theta }\sin\theta )Ile^{il\phi }$. When *kr* ≫ 1, the electric field distribution is expanded as

(6) E¯nr¯,ϕiωμI¯¯−r^r^·eikr4πrf¯θ,ϕ=−θ^iωμIleikr4πrsin⁡θeilϕ

The electric field distribution in transverse* xy*-plane at* z* = 0 can be viewed as a superposition of arrayed electric dipoles as

(7) $$\begin{array}{c} {\bar{E}}_{total}\left(\;\bar{r},\phi \;\right)=\overset{\infty }{\underset{n=-\infty }{\sum }}{\bar{E}}_{n}\left(\;\bar{r},\phi \;\right)=-\hat{z}\left(\;A\frac{e^{ikr}}{4\pi r}e^{il\phi }\;\right) \end{array}$$

where* A* is amplitude. Numerical results obtained from (7) show the same spiral pattern as those in simulation (Figure 2). Electric field and phase distributions for the left-handed helical metagrating are plotted in Figures 3(b)-3(c). A clear spiral pattern is generated with a phase accumulation of -2*π* in the *ϕ* direction, indicating an angular momentum of* l* = -1. The existence of complex-valued solutions was discussed by Mittra [41]. More details can be found in the Supplementary Information ([Supplementary-material supplementary-material-1]).

**Figure 4:**
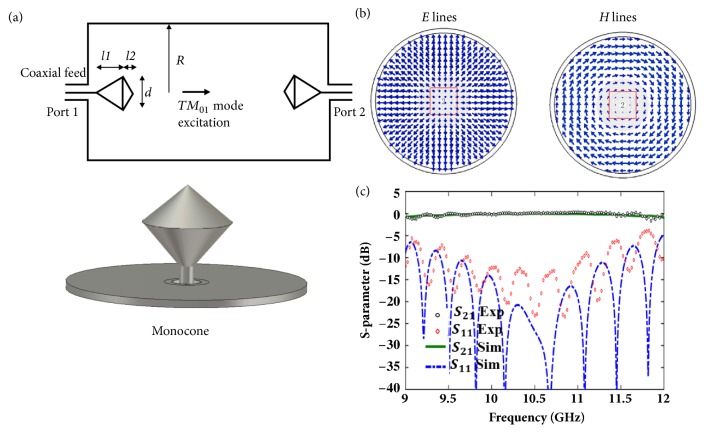
Designer* TM*_*01*_ mode transducer and S-parameters. (a) Schematic of a monocone antenna transducer. (b) Field pattern of the* TM*_*01*_ mode in the circular waveguide. (c) Simulated and measured S-parameters.

Experiment is performed to verify the spiral field generation in Smith-Purcell radiation in the microwave regime. A guided wave in the circular waveguide is utilized to mimic the field generated by moving electron beams. There are several principles to design the waveguide mode. First,* TM*_*01*_ is chosen because of its radial diverging electric field and azimuthally symmetric magnetic field loops. The field patterns are similar to those of a moving electron. Then the circular waveguide is filled with a dielectric to increase the propagating constant and thus excite evanescent waves at the boundary to mimic the moving electron. The details can be found in the Supplementary Information. Second, a monocone antenna is designed to excite second dominant* TM*_*01*_ mode while blocking first dominant* TE*_*11*_ mode and all other higher modes in the circular waveguide. The monocone antenna (Figure 4(a)) at the input of circular waveguide acts as a* TM*_*01*_ mode transducer. A 50Ω coaxial connector is used to feed the power. The dimensions are optimized to achieve maximum conversion efficiency and bandwidth. The optimized geometries of the* TM*_*01*_ mode transducer are* l*_*1*_ = 7* mm*,* l*_*2*_ = 5* mm*, and* d* = 12* mm*. The radius of the circular waveguide is* R* = 15mm. Full-wave simulations (CST Microwave Studio) have been performed to evaluate the efficiency of* TM*_*01*_ transducer. The electric, magnetic lines and S-parameters of the monocone transducer are illustrated in Figures 4(b) and 4(c), respectively. Conversion efficiency is larger than -0.5 dB over 9.1-11.9 GHz and the reflection coefficient is smaller -10 dB over 9.4-11.5 GHz. The measured transmission and reflection coefficients are plotted in Figure 4(c), in good agreement with simulated results.

**Figure 5:**
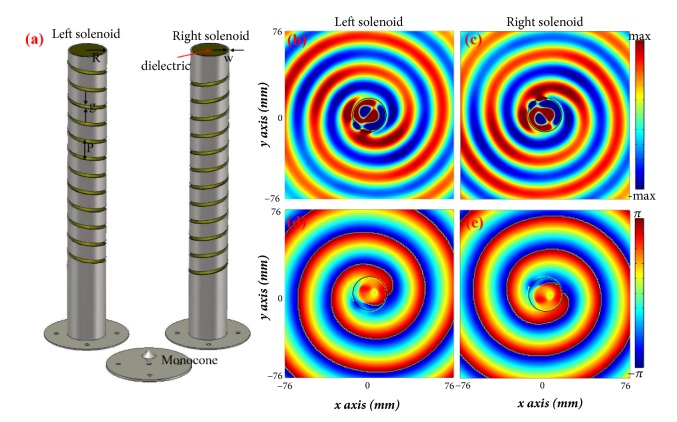
Spiral field Smith-Purcell radiation generators and simulation results. (a) Schematic of two spiral field Smith-Purcell radiation generators of opposite handedness. (b) Scattered* E*_*z*_ field distribution and (d) phase distribution for the left-handed generator. (c) Scattered* E*_*z*_ field distribution and (e) phase distribution for the right-handed generator. The working frequency is 10 GHz.

The designed two spiral field Smith-Purcell radiation generators are shown in Figure 5(a). Each generator consists of a helical metagrating at the boundary, a monocone transducer at the bottom and the dielectric filler. The distance between the monocone transducer and the first helical unit is 110* mm*, in order to reduce the direct coupling between monocone transducer and helical structures. The optimized gap size of the helical metagrating is* g* = 3* mm*, with width* w* = 1* mm* and helix pitch* p* = 15* mm* and these parameters are different values in microwave regime from these in terahertz. FR4 is selected as the dielectric filler with the relative permittivity of 4.5 and the loss tangent of 0.025. Full-wave simulations (CST Microwave Studio) have been performed to verify the spiral field generation in Smith-Purcell radiation. For the left-handed generator, the* E*_*z*_ field distribution and its phase response are plotted in Figures 5(b) and 5(d) corresponding to the angular momentum* l *= -1. At the meantime, for the right-handed one, spiral pattern is generated in the opposite direction as illustrate in Figures 5(c) and 5(e), corresponding to the angular momentum* l *= +1. As expected, the verification of the spiral field Smith-Purcell radiation in the microwave regime agrees well with the real electron case.

**Figure 6:**
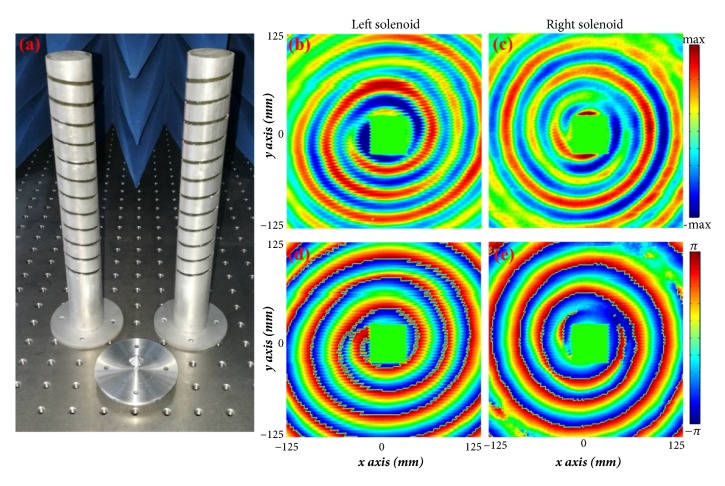
Fabricated spiral field Smith-Purcell generators and the measured results. (a) Photograph of the helical metagratings and the monocone transducer. (b) Measured radiated* E*_*z*_ field distribution and (d) phase distribution for the left-handed generator. (c) Measured radiated* E*_*z*_ field distribution and (e) phase distribution for the right-handed generator. The working frequency is 10 GHz.

To experimentally demonstrate the spiral field generation in Smith-Purcell behavior, the helical metagratings and monocone transducer have been fabricated by laser beam cutting technology.  Figure 6(a) shows the photograph of the samples, with the same size as the simulated ones. The field distributions have been measured in an anechoic chamber. The experimental setup includes an Agilent Network Analyzer and a two-dimensional near-field scanning platform. The scanning area is 250 mm × 250 mm and the probe position at center of helical metagrating plane is automatically controlled by an electric motor and a software program. The measured radiated* E*_*z*_ electric field distributions for the two helical metagratings are plotted in Figures 6(b) and 6(c), respectively. Their corresponding phase distributions are plotted in Figures 6(d) and 6(e). One can observe opposite the spiral radiated field with angular momenta (*l* = ±1). As expected, the measured results agree well with the theoretical prediction and the spiral field generation in Smith-Purcell radiation is demonstrated in the microwave regime using circular waveguides.

## 3. Conclusions and Outlook

In conclusion, the interaction between swift electrons and helical metagratings have been proposed and experimentally verified. Spiral field patterns are generated in Smith-Purcell radiation when moving electrons pass through the helical metagratings. The angular momentum direction of the spiral field Smith-Purcell radiation is only dependent on the helicity of metagratings. Semitheoretical analysis of the spiral field generation in Smith-Purcell radiation from a helical metagrating is also performed. The intrinsically nonradiative energy bound at the source current sheet is coupled to the electric dipole with an azimuthal phase factor when swift electrons pass through the helical metagrating. Subsequently, two generators for controlling over spiral field Smith-Purcell radiation have been constructed by a combination of helical metagratings, a monocone transducer and dielectric fillers. Measured field patterns from near-field scanning platform verify the theoretical predictions on spiral field Smith-Purcell radiation in the microwave regime. These findings may provide an approach to understand the interplays between electrons, photons, and chiral objects. Our work can significantly promote the advance in novel electron microscopy techniques and electron-beam-based photonic technologies as well as the emerging area of photonic spin-orbit interactions.

## Supplementary Materials

Supplementary Materials Figure S1: the equivalent process of an electric dipole with an azimuthal factor. Figure S2: (a) a circular metallic waveguide; (b) *k*_*z*_*a*-*ka* diagram for the guided modes; and (c) field lines for* TM*_*01*_ mode. Figure S3: (a) a moving charged particle; (b) a phased dipole array composed of an infinite number of dipoles along the z axis; and (c) evanescent wave electric field distribution.
