# Supplementary material for: Spiral Field Generation in Smith-Purcell Radiation by Helical Metagratings
Source: Research (Wash D C). 2019 Feb 27;2019:3806132. doi: 10.34133/2019/3806132 (PMC6750070; doi:10.34133/2019/3806132)
Supplement: Supplementary Materials — Figure S1: the equivalent process of an electric dipole with an azimuthal factor. Figure S2: (a) a circular metallic waveguide; (b) kza-ka diagram for the guided modes; and (c) field lines for TM01 mode. Figure S3: (a) a moving charged particle; (b) a phased dipole array composed of an infinite number of dipoles along the z axis; and (c) evanescent wave electric field distribution. [file 3806132.f1.docx]

Supporting Information for

Spiral field generation in Smith-Purcell radiation by helical metagratings

Liqiao Jing^1,3^†, Zuojia Wang^2^†^*^, Xiao Lin^4^, Bin Zheng^1^, Su Xu^5^, Lian Shen^1^, Yihao Yang^4^, Fei Gao^1^, Min Chen^3^, Hongsheng Chen^1*^

^1^Key Lab. of Advanced Micro/Nano Electronic Devices & Smart Systems of Zhejiang, College of Information Science and Electronic Engineering, Zhejiang University, Hangzhou 310027, China.

^2^School of Information Science and Engineering, Shandong University, Qingdao 266237, China

^3^Department of Physics, Massachusetts Institute of Technology, Cambridge, Massachusetts 02139, USA

^4^Division of Physics and Applied Physics, School of Physical and Mathematical Sciences, Nanyang Technological University, Singapore, Singapore

^5^State Key Laboratory of Integrated Optoelectronics, College of Electronic Science and Engineering, Jilin University, Changchun 130012, China.

† Co-first authors.

^*^Corresponding authors:

(Z. Wang) z.wang@sdu.edu.cn, (H. Chen) hansomchen@zju.edu.cn

1. **Equivalent an electric dipole with an azimuthal factor**

When a swift electron moves through a helical metgrating, the current will be induced along the gap of the helix metasurface as shown in the left of Fig. S1. In our design, the velocity of the moving electron is c/8 and the periodicity of the helical metagrating is $\lambda_{0}/8$. When the swift electron passes through one periodicity, the induced dipoles have a phase change 2$\pi$. Therefore, the helical dipole array can be equivalent to a circular dipole array with an azimuthal phase factor. Due to the periodicity of the helical metagrating can be neglected compared to the wavelength ($p<<\lambda_{0}$). Finally, the circular dipole array with phase factor can be equivalent an electric dipole with an azimuthal factor as shown in right of Fig. S1.


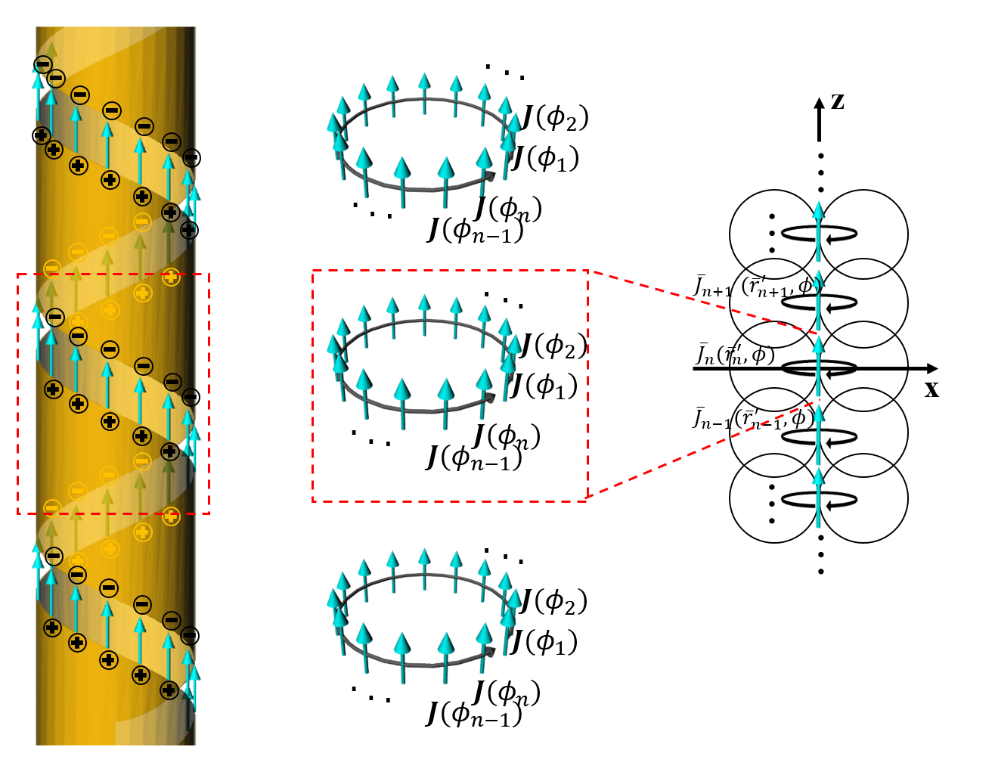


**Figure S1.** The equivalent process of an electric dipole with an azimuthal factor.

**2. The heliccal metagrating design in microwave regime**

Consider a circular metallic waveguide with radius as shown in Fig. S2(a). The boundary conditions require that $E_{z}$ and vanish at $\rho=a$. For TM waves, we have

$E_{z}=J_{m}(k_{\rho}\rho)\left\{ \begin{matrix} \sin m\phi\\ \cos m\phi\end{matrix} \right\}e^{ik_{z}z}$ (S1)

$\begin{matrix} E_{\rho}=\frac{ik_{z}k_{\rho}}{\omega^{2}\mu\varepsilon-k_{z}^{2}}J_{m}^{'}(k_{\rho}\rho)\left\{ \begin{matrix} \sin m\phi\\ \cos m\phi\end{matrix} \right\}e^{ik_{z}z} \\ \end{matrix}$ (S2)

$\begin{matrix} E_{\phi}=\frac{ik_{z}}{\omega^{2}\mu\varepsilon-k_{z}^{2}}\frac{m}{\rho}J_{m}(k_{\rho}\rho)\left\{ \begin{matrix} \cos m\phi\\ -sinm\phi\end{matrix} \right\}e^{ik_{z}z} \\ \end{matrix}$ (S3)

$\begin{matrix} H_{\rho}=\frac{-i\omega\varepsilon}{\omega^{2}\mu\varepsilon-k_{z}^{2}}\frac{m}{\rho}J_{m}(k_{\rho}\rho)\left\{ \begin{matrix} \cos m\phi\\ -sinm\phi\end{matrix} \right\}e^{ik_{z}z} \\ \end{matrix}$ (S4)

$\begin{matrix} H_{\phi}=\frac{i\omega\varepsilon k_{\rho}}{\omega^{2}\mu\varepsilon-k_{z}^{2}}J_{m}^{'}(k_{\rho}\rho)\left\{ \begin{matrix} \sin m\phi\\ \cos m\phi\end{matrix} \right\}e^{ik_{z}z} \\ \end{matrix}$ (S5)

with the dispersion relation

$\begin{matrix} k_{z}^{2}+k_{\rho}^{2}=\omega^{2}\mu\varepsilon\\ \end{matrix}$ (S6)

The boundary condition of vanishing $E_{z}$ and at $\rho=a$ gives the guidance condition

$J_{m}(k_{\rho}a)=0$ (S7)

From the guidance condition and dispersion relation, we find

$\begin{matrix} k_{z}=\sqrt{\omega^{2}\mu\varepsilon-\left( {\xi_{mn}}/a \right)^{2}} \\ \end{matrix}$ (S8)

$\xi_{mn}$ is the *n*th root of the *m*th order Bessel function. The *k_z_a-ka* diagram for the guided *TM_mn_* modes is plotted in Fig. S2(b). We find from Fig. S2(b) that for the *TM_01_* mode $\xi_{01}$ = 2.4. The field patterns for *TM_01_* mode is shown Fig. S2(c) which is similar with the field patterns of a moving electron. To satisfy the condition for spiral field Smith-Purcell radiation in Eq. (3), high permittivity dielectric should be filled into the circular waveguide. FR4 is selected as the dielectric with the relative permittivity of 4.5 and the loss tangent of 0.025. The radius of the circular waveguide is set 15 *mm*. According to Eq. (S8), the propagation wave number in z direction is $k_{z}=\sqrt{\omega^{2}\mu\varepsilon-(\xi_{mn}/a)^{2}}=2k_{0}$ at working frequency 10 GHz. Therefore, the equivalent electron velocity $v=c/2$.


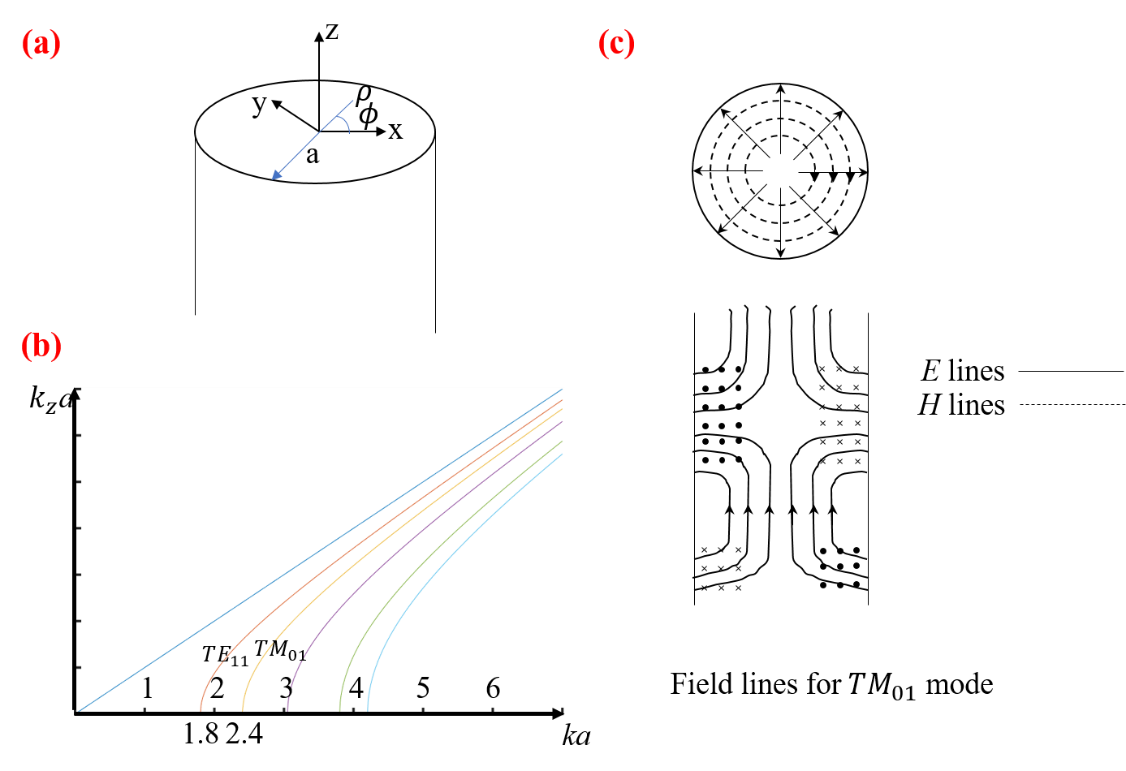


**Figure S2.** (a) A circular metallic waveguide. (b) *k_z_a-ka* diagram for the guided modes and (c) field lines for *TM_01_* mode.

A charged particle q moves along the *z*-axis with the velocity v as shown in Fig. S3(a), the current density is

$\bar{J}_{p}\left( \bar{r},t \right)=\bar{z}qv\frac{\delta\left( \rho\right)}{2\pi\rho}\delta(z-vt)$ (S9)

Through the Fourier transform, we obtain the expression of this current in frequency domain as

$\bar{J}_{p}\left( \bar{r},\omega\right)=\bar{z}\frac{q}{4\pi^{2}\rho}\delta(\rho)e^{ik_{z}z}$ (S10)

Here, .

When a phased dipole array is adopted to model the charged particle, we assume the dipole array works at the frequency $\omega_{0}$ as shown in Fig. S3(b). The current density of the nth dipole is

$\bar{J}_{n}\left( \bar{r},t \right)=\bar{z}Il\frac{\delta\left( \rho\right)}{2\pi\rho}e^{in\alpha-i\omega_{0}t}$ (S11)

The total current density of all the dipoles is

$\bar{J}_{d}\left( \bar{r},t \right)=\bar{z}Il\frac{\delta\left( \rho\right)}{2\pi\rho}\sum_{-\infty}^{+\infty} \delta\left( z-n\Delta\right)e^{in\alpha-i\omega_{0}t}$ (S12)

The current density of the dipole array in the frequency domain can be calculated by the Fourier transform:

$\bar{J}_{d}\left( \bar{r},\omega\right)=\bar{z}Il\frac{\delta\left( \rho\right)}{2\pi\rho}\delta(\omega-\omega_{0})\sum_{-\infty}^{+\infty} \delta\left( z-n\Delta\right)e^{in\alpha}$ (S13)

Assuming $\alpha=k_{z}\Delta$ and $\Delta=l$, the current density of the dipole array becomes

$\bar{J}_{d}\left( \bar{r},\omega\right)=\bar{z}\frac{I\delta\left( \omega-\omega_{0} \right)}{2\pi\rho}\delta(\rho)e^{ik_{z}z}$ (S14)

At the operating frequency $\omega_{0}$, the behavior of the moving charged particle and the phased dipole array will be exactly equivalent. Therefore, we use a waveguide with an array of open slots to model a moving charged particle.

Then we fill the dielectric into the waveguide to make sure an equivalent velocity v=0.5c to satisfy the necessary conditions of helix pitch. Fig. S3(c) shows the evanescent wave when the waveguide is filled with dielectric.


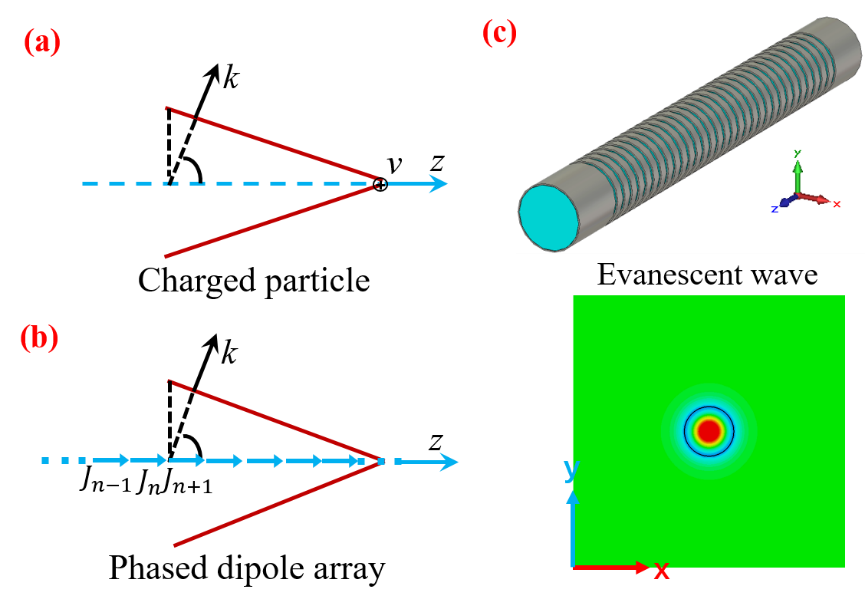


**Figure S3.** (a) A moving charged particle and (b) a phased dipole array composed of an infinite number of dipoles along the z axis. (c) evanescent wave electric field distribution.
